# Dissecting the genetic variation of haploid frailty in maize for enhanced doubled haploid breeding

**DOI:** 10.3389/fpls.2025.1646128

**Published:** 2025-10-07

**Authors:** Mercy Fakude, Ann Murithi, Yu-Ru Chen, Recep Yavuz, Siddique Imran Aboobucker, Ursula Karoline Frei, Thomas Lübberstedt

**Affiliations:** Department of Agronomy, Iowa State University, Ames, IA, United States

**Keywords:** haploid frailty, haploid vigour, haploids, diploids, doubled haploid breeding, perfect isogenic haploid–diploid pairs, haploid male fertility, haploid female fertility

## Abstract

Haploid frailty (HF), the reduced vigour of haploids compared to their diploid counterparts, limits the efficiency of doubled haploid (DH) breeding in maize. This study evaluated 192 BS39 perfect isogenic haploid–diploid pairs across two replications to quantify HF in eight agronomic traits and integrate analyses of HF with haploid fertility, genomic prediction, and genome-wide association studies (GWAS). Haploids were on average 30–40% shorter in plant and ear height, while traits such as tassel branch number showed minimal or even negative frailty, indicating the presence of naturally vigorous haploids. Heritability was consistently high for diploid (0.8–0.9) and haploid performance (0.7–0.9), but lower for HF (0.1–0.7), reflecting its complexity as a derived trait. Correlation analyses revealed strong diploid–haploid relationships (r = 0.5–0.9) and moderate negative associations between haploid performance and HF (r = –0.4 to –0.6). Importantly, haploid female fertility correlated positively with vigour traits, suggesting that dual improvement of vigour and fertility is possible. Genomic prediction using diploid data showed moderate accuracy for haploid traits (0.3–0.5) but weak performance for HF, showing the need for direct haploid evaluations. GWAS detected significant SNPs across traits, including 14 for plant height, 30 for ear height, 20 for flag leaf length, 19 for flag leaf width, 23 for tassel length, 2 for spike length, and 6 for tassel branches, with one SNP shared between haploid and diploid tassel branches. Candidate genes included regulators of cell cycle control (Myb3R-1), auxin signalling (SRS1, SAUR-like proteins), cytoskeletal organization (PRONE-domain proteins), and oxidative stress protection (HGGT1).

## Introduction

Maize (*Zea mays* L.) is one of the world’s most important cereal crops, serving as a staple food for billions of people and a vital source of animal feed and industrial products (Nuss and Tanumihardjo, 2010). To meet the increasing demand for maize and address challenges posed by climate change and evolving pest pressures, continuous improvement of maize varieties is essential (Lobell and Field, 2007). Doubled haploid (DH) breeding is an alternative to conventional maize breeding methods (Santos et al., 2022). DHs offer accelerated development of homozygous lines in two generations (Boerman et al., 2020). Despite the potential of DH technology to accelerate breeding progress, the phenomenon of haploid frailty presents a significant barrier in the DH protocol. Chase (1964) hypothesized that haploid maize plants, having half the chromosomal volume of diploid plants, would be proportionally smaller but still possess the same number of leaves and branches. However, his observations contradicted this assumption. Haploid plants not only exhibited reduced overall size but also had fewer leaves and branches than their diploid counterparts. Mature haploids were approximately 70% as tall as diploids, with a leaf area around 56% of that of their diploid counterparts. These findings provided the first documented evidence of haploid frailty, illustrating that reduced vigor in haploids is accompanied by both smaller size and a reduced number of plant parts.

Haploid frailty (HF) manifests as reduced vigor and inferior performance in haploid plants compared to their diploid counterparts, limiting the practical application of DH technology in breeding programs. HF is calculated as a relative reduction of the performance of haploids compared to isogenic diploid plants. High HF percentages indicate a substantial difference in the relative haploid vs. diploid performance. Negative HF percentages would suggest an unexpected but potentially advantageous scenario where haploid plants excel in specific traits. Generally, more vigorous haploid plants would help to fully leverage DH technology by ensuring successful production of DH (D1) lines from initial haploid (D0) plants, which is a limiting factor of successful DH line production. Moreover, if more seed could be produced on D0 plants, then it would allow early per se testing and testcross seed production, speeding up the breeding process.

Two recent studies have clarified complementary aspects of haploid biology. Fakude et al. (2025) demonstrated that spontaneous haploid genome doubling (SHGD) can replace colchicine treatment by restoring haploid fertility. Using BS39-derived haploid isogenic lines, they mapped loci associated with haploid female fertility and haploid male fertility and identified candidate genes related to cytoskeleton dynamics, cell cycle regulation, and hormone signaling. In parallel, Yavuz et al. (2025) quantified haploid frailty across eight agronomic traits, estimated trait heritability, and showed that the *qshgd1* locus reduces frailty and enhances vigor, thereby emphasizing SHGD’s role in restoring vigour. Collectively, these studies revealed the genetic basis of fertility restoration and vigor improvement through SHGD, but did not integrate frailty, fertility, genome-wide association and candidate gene search into a single framework.

The present study builds directly on this foundation and aims to provide phenotypic and genetic findings relevant to overcoming haploid frailty and enhancing the efficiency of DH breeding. Working with the same panel of 192 haploid isogenic lines (HILs) and their corresponding 192 isogenic inbred lines from Yavuz et al. (2025), the present study quantified haploid frailty across eight agronomic traits, identifying haploid lines with unexpectedly high vigor, particularly for plant and ear height; (ii) tested correlations among diploid performance, haploid performance, and HF% to evaluate predictive relationships; (iii) evaluated the association between frailty traits and HFF/HMF to determine whether haploid vigour is linked to haploid fertility restoration; and (iv) conducted a comprehensive GWAS for diploid, haploid, and HF%, followed by candidate gene analysis. By combining phenotypic evaluation of haploid frailty, fertility assessment, and association mapping within the same panel, the present study represents, to the best of our knowledge, the first comprehensive analysis of haploid frailty and fertility in maize.

## Materials and methods

### Plant materials

The BS39 population was derived from five exotic Tusón accessions and has been adapted to the US Midwest photoperiod conditions through several generations of intercrossing (Hallauer and Carena, 2016). BS39-SHGD-DH lines were derived from a cross between BS39 and inbred A427 (Verzegnazzi et al., 2021). Inbred A427 (~ 78% HMF) was used as a SHGD donor. Both BS39 and BS39-SHGD-derived DH lines were genotyped by genotyping-by-sequencing (GBS) (Verzegnazzi et al., 2021). In summer 2022, we induced 228 inbred lines, 85 derived from the original BS39 population (BS39-inbreds) and 143 inbred lines derived from the cross between BS39 and the SHGD-donor inbred A427 (BS39-SHGD-inbreds). The 228 inbred lines were crossed to an inducer line (BHI306) to produce haploid seed. After harvesting, haploid selection was done manually based on the expression of *R1-Navajo* in the embryo and aleurone. After haploid selection, we obtained haploid isogenic lines (HILs) for 192 of the 228 inbreds, 66 BS39-HILs, and 126 BS39-SHGD-HILs.

### Experimental design

In the summer of 2023, the 192 HILs were planted side by side with their corresponding 192 isogenic inbred lines. The experiment followed a randomized complete block design, with each line planted with 20 seeds at a within-row spacing of 10 cm and a between-row spacing of 0.7 m. This experiment was undertaken in two replications (REP1 and REP2), with REP1 planted 11 days before REP2, at the Agricultural Engineering and Agronomy Research Farm, Boone, Iowa, USA. Although conducted at the same physical site, the 11-day planting gap exposed the two replications to different conditions during critical growth stages, creating distinct temporal environments. For analysis, REP1 and REP2 were therefore treated as two environments for the computation of BLUES and subsequent GWAS. During growth, misclassified haploids (i.e., hybrids) were identified and removed from the field from the V2 through V6 growth stages. Classification was based on visual appearance, particularly increased vigour and larger plant and leaf sizes compared to the short, narrow, and upright leaf morphology typical of true haploids (Aboobucker et al., 2022; Dermail et al., 2024).

### Phenotypic data acquisition

Eight agronomic traits were evaluated: plant height (PH), ear height (EH), stem diameter (SD), flag leaf length (FLL), flag leaf width (FLW), tassel length (TL), tassel branch number (TB), and tassel spike length (SL), along with haploid female fertility (HFF) and haploid male fertility (HMF). These traits capture key aspects of vegetative vigor, canopy architecture, reproductive potential, and fertility (Hallauer and Miranda Filho, 1988; Duvick, 2005; Tollenaar and Lee, 2006; Setimela et al., 2018; Liu et al., 2023; Fonseca et al., 2023).

Data collection for the eight agronomic traits followed the standardized protocols outlined in the Maize Handbook (Freeling and Walbot, 1994). Haploid frailty was computed for each trait as the relative reduction in haploid performance compared to its isogenic diploid counterpart. For example, haploid frailty for plant height was calculated as:


$$\text{Percentage haploid frailty for plant height}=[\frac{\text{diploid plant height }-\text{ haploid plant height}}{\text{diploid plant height}}]\times 100$$

This approach was applied analogously to the other traits, enabling quantification of trait-specific reductions in haploid performance relative to diploids. Trait measurements were conducted for both diploid and haploid lines. Plant height was measured at the VT and R1 stages from ground level to the tip of the tassel using a measuring ruler. Ear height was measured at the VT and R1 stages from ground level to the base of the ear using a measuring tape. Stem diameter was measured at the R2 stage using a caliper placed around the second internode from the bottom. Flag leaf length was measured at the R2 stage using a tape ruler from the leaf collar to the tip of the leaf. Flag leaf width was measured at the R2 stage at the widest point of the leaf using a measuring tape. Tassel length was measured at the R2 stage from the base to the tip of the tassel using a measuring tape. Spike length was measured at the R2 stage from the base to the tip of the spike. Number of tassel branches were counted at the R1 stage by recording the number of lateral branches that emerged from the central spike.

Data for haploid male fertility (HMF) percentage was collected at flowering. HMF percentage was calculated by dividing the number of pollen-shedding haploids by the total number of haploids in each plot as follows:


$$HMF(\%)=[\frac{\text{number of pollen shedding haploids }}{\text{total number of haploids }}]\times 100$$

Data for haploid female fertility (HFF) was counted and recorded at harvest, as the average number of kernels per plot. To adjust the average number of kernels per plot, final plant stand was counted, and the average number of kernels per plot was computed as follows:


$$\text{Average}\;HFF=[\frac{\text{number of kernels per plot }}{\text{total number of haploids per plot}}]$$

### Statistical analysis of phenotypic data

Linear models were fitted for all eight traits in R using the *lme4* package (Bates et al., 2015) as follows:


Yij=µ+Gi+Ej+ϵij

Where *Yij* is the observed diploid performance, haploid performance and (computed) haploid frailty percentage on the *i*-th genotype (*i =* 1, 2,…,192*)* and *j*-th environment (*j* = 1,2), *µ* is the overall mean, *Gi* is the fixed effect of the *i*-th genotype, *Ej* is the fixed effect of the *j*-th environment and *εij* is the random error.

Violation of normal distribution for computed haploid frailty, haploid performance, and diploid performance data was evaluated using Shapiro-Wilk tests in R software (Shapiro and Wilk, 1965). When the p-value was <0.05, then transformation was performed on traits using the ‘*bestNormalize*’ package in R. Analysis of variance (ANOVA) for computed haploid frailty was performed after the traits were transformed. Transformation was performed on data for all eight traits, as their distributions did not follow a normal distribution. Heritability on an entry mean basis for the eight traits was computed as:


$$h^{2}\;=\frac{\sigma_{\epsilon }^{2}}{\sigma_{\epsilon }^{2}\;+\;\frac{\sigma_{\epsilon }^{2}}{n}\;}\;$$

Where $\sigma_{\epsilon }^{2}$ is the genetic variance component, $\sigma_{\epsilon }^{2}$ is the variance component for random error, and n is 2. Traits with low heritability estimates either on their diploid level, haploid level, and haploid frailty percentage were excluded from correlation computations. Thus, correlations between diploids and haploids (D-H) were performed for only seven traits excluding stem diameter. Correlations between haploids and haploid frailty (H-HF) and correlations between diploids and haploid frailty (D-HF) were computed for four traits, excluding stem diameter, flag leaf length, flag leaf width, and number of tassel branches. Given the critical role of haploid male fertility and haploid female fertility in DH production (Fakude et al., 2025), we further explored their correlation with the eight agronomic traits examined in this study. Thus, a correlation matrix including HMF, HFF, and the eight traits at their diploid and haploid level and their haploid frailty percentage was computed. All correlations were computed using Spearman correlation in R. The pooled data from the two replications were used to compute Best Linear Unbiased Estimators (BLUEs) for diploid performance, haploid performance, and haploid frailty percentage in R using the *lme4* package. These BLUEs served as the phenotypic data for the genome-wide association study (GWAS).

### Genomic prediction

The 192 genotypes were divided into the training set and the test set. The agronomic traits of diploids in the training set were used to calibrate the genomic prediction model. The predictive ability was the Pearson correlation coefficient of predicted values of the traits of diploids and the BLUEs of diploids and haploids in the test set, which was estimated on average by five-fold cross-validation with five replicates.

### Genomic prediction models

The prediction models were built using ridge regression BLUP (rrBLUP), executed via the rrBLUP package in R (Endelman, 2011). The predictions were generated by estimating the effects of genome-wide SNPs. The model is expressed as:


y= μ+Zu+ϵ

Where y represents the vector of BLUE values for a single trait, μ is the overall mean. Z is the incidence matrix of genome-wide SNP genotypes, and u is a vector of random SNP effects, assuming $u\sim N(0,I\sigma_{m}^{2})$ where I is the identity matrix and $\sigma_{m}^{2}$ is the SNP marker variance. The residual error vector ϵ follows $\epsilon \sim N(0,\;I\sigma_{e}^{2})$ .

### Genotyping and SNP calling

Genotyping and SNP calling were previously performed by Verzegnazzi et al. (2021) and Santos et al. (2022). Genotyping by Sequencing (GBS), as described by Elshire et al. (2011), was conducted for 471 lines, comprising 153 BS39-DHs and 318 BS39-SHGD-DHs. This process yielded a total of 955,690 SNPs. For the present study, the genotypic data of the 192 DH lines were sampled from this larger dataset of 471 lines. Filtering of the SNP data was conducted using TASSEL 5.2.58 (Glaubitz et al., 2014). SNPs with a minor allele frequency (MAF) below 5% and a call rate below 50% were excluded. Additionally, lines exhibiting more than 5% heterozygosity were discarded. Any remaining heterozygous loci were considered as missing data. After applying these filtering criteria, 414,337 SNPs were retained for the 66 BS39-DHs and 126 BS39-SHGD-DHs that were used to genotype 192 HILs. The selected SNPs were annotated to represent the corresponding chromosome number and base pair position.

### Linkage disequilibrium and principal components analysis

Linkage disequilibrium decay, kinship, and principal component analysis were performed in R software using the GAPIT package (Tang et al., 2016). A genome-wide LD was quantified using the squared coefficient of correlation (R^2^) values of alleles, which provided insights into the non-random association of alleles at different loci. A kinship matrix was computed using the VanRaden method (VanRaden, 2008) in GAPIT. A principal component analysis (PCA) was performed to account for population structure, relatedness, and dimensionality. By performing PCA on the genotype data matrix, GAPIT identified the major axes of genetic variation, represented by principal components (PCs), which capture distinct patterns of genetic diversity within the population. The top three PCs, explaining the most genetic variation, were interpreted as indicators of population structure. These PCs served as covariates in the subsequent association mapping analyses.

### Genome-wide association mapping

GAPIT was used for the association between SNP genotypes markers and agronomic traits of diploids, haploids, and haploid frailty percentages. Association mapping was only performed for traits showing significant genetic variation in the analysis of variance (ANOVA). FarmCPU was selected based on its ability to integrate genetic relatedness as a random effect to account for population structure (Liu et al., 2016). The Simple-M method (Gao et al., 2008) was used for multiple hypothesis correction to control the family-wise error rate. Simple-M estimates the effective number of independent tests by accounting for linkage disequilibrium among SNPs, providing an adjusted significance threshold that is less conservative than the traditional Bonferroni correction.

### Mapping of potential candidate genes

Candidate gene search was performed for agronomic traits with highly significant SNPs and SNPs overlapping across diploids, haploids and haploid frailty percentages. Candidate genes were identified within the 200 kb regions upstream or downstream of stable QTL loci, utilizing the B73 RefGen_v5 reference genome in MaizeGDB (http://www.maizegdb.org). To align SNP data developed using the B73v2 reference genome with the updated B73v5 reference genome, a coordinate conversion for the flanking regions surrounding each SNP was conducted. Approximately 200 kb both upstream and downstream of each SNP were considered. The conversion process involved identifying a gene at the boundary of each 200 kb segment in the B73v2 genome and confirming its updated location in the B73v5 genome. This approach enabled the accurate redefinition of the SNP-flanking regions based on the positional shifts of these boundary genes in the newer genome version. Genes found directly on or close to each associated SNP were considered possible candidate genes for the traits.

## Results

### Trait distributions of haploids, diploids, and haploid frailty percentages

**Figure 1:**
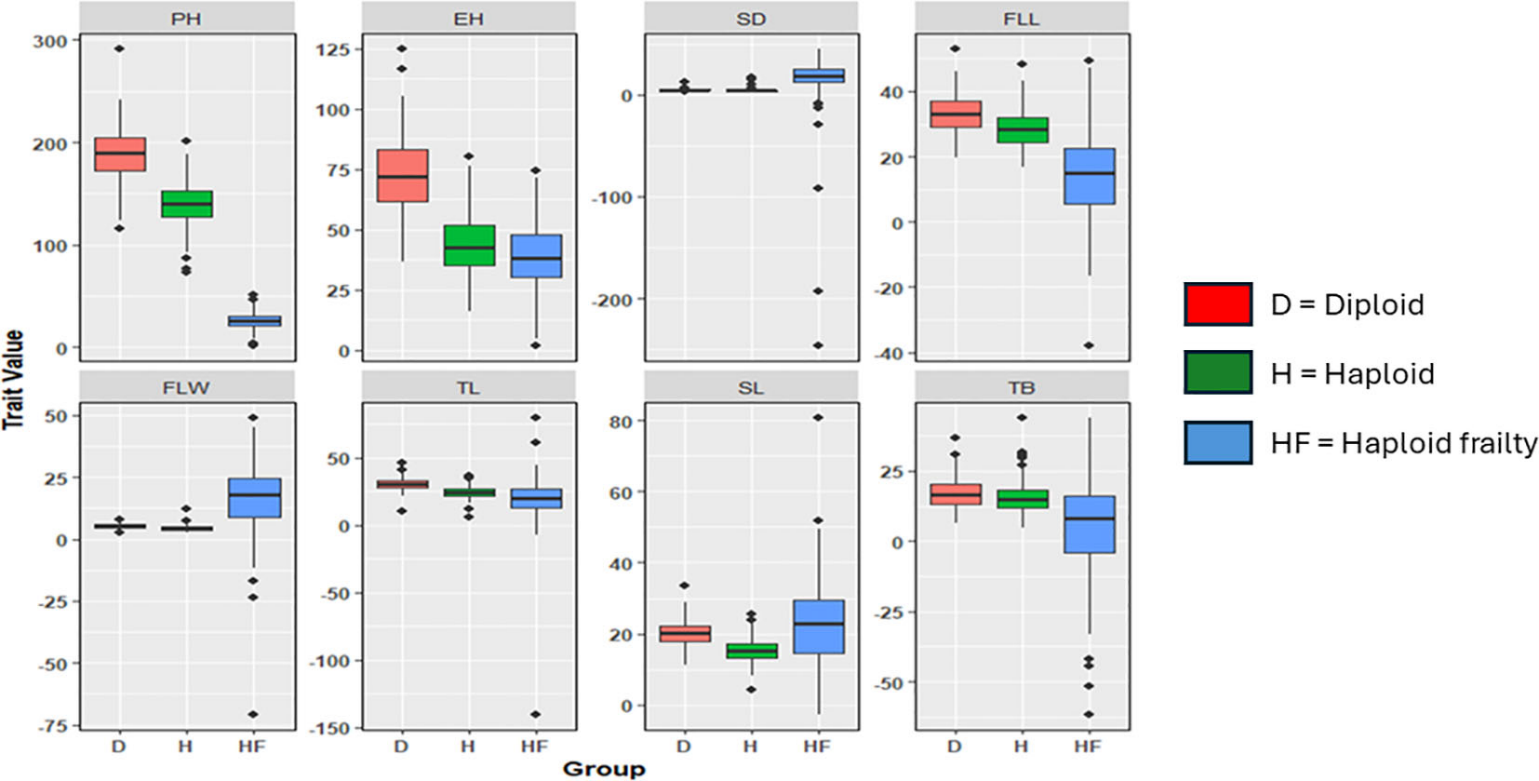
Distribution of diploid performance, haploid performance, and haploid frailty %. PH, Plant height; EH, Ear height; SD, Stem diameter; FLL, Flag leaf length; FLW, flag leaf width; TL, Tassel length; SL, spike length; TB, number of tassel branches.

The boxplots revealed varying degrees of overlap between diploid performance, haploid performance, and haploid frailty percentages across the different agronomic traits. For instance, the median plant height for diploids was around 190 cm, while haploids showed a reduction to about 140 cm, yet the interquartile range (IQR) overlap suggested that certain haploid individuals reached similar heights. A similar pattern was observed for ear height, where the diploid median was above 70 cm compared to around 40 cm for haploids, but overlap in the IQR indicates that some haploids achieve comparable heights. In terms of stem diameter, diploids had a median of around 3 cm. Although haploids generally showed thinner stems (2 cm), there was an overlap that suggested that some haploids achieved near-diploid thickness. This pattern was consistent for flag leaf length, flag leaf width, tassel length, spike length, and number of tassel branches, where diploids typically outperformed haploids. However, the overlap in the IQRs implied that not all haploids were equally frail. Notably, number of tassel branches showed the highest overlap, with a median of 18 branches for diploids and 17 branches for haploids (**Figure 1**).

### Statistical summary and heritability of agronomic traits

Diploids exhibited a mean plant height of 188.7 cm, with a maximum of 291.7 cm. Haploids showed a lower mean of 139.4 cm with a maximum of 200.8 cm. This trend was consistent with other traits like ear height, where diploids had a mean of 72.4 cm, compared to 43.9 cm in haploids, and stem diameter, with diploids at 4.8 cm versus 4.1 cm in haploids. Haploid frailty percentages generally reflect these reductions, with a mean haploid frailty of 30% for plant height and 40% for ear height, demonstrating significant decreases in haploid performance. However, the number of tassel branches, was similar for haploids and diploids (15.6 and 16.8), respectively, with haploid frailty percentages ranging from a minimum of -60% to 40% (**Supplementary Figure S1**).

Heritability across different traits revealed distinct trends for diploid performance, haploid performance, and haploid frailty percentages. Diploids consistently exhibited the highest heritability, with values ranging from 0.8 to 0.9 for all traits, except stem diameter (0.2). In contrast, haploids generally showed slightly reduced heritability compared to diploids, with values in the range of 0.7 to 0.9, except for stem diameter (0.1). Haploid frailty consistently showed the lowest heritability estimates compared to both diploids and haploids, with values of 0.6-0.7 for plant height, ear height, and spike length, and 0.1-0.3 for stem diameter, flag leaf length, flag leaf width, tassel length and tassel branches (**Supplementary Figure S2**). Overall, haploid and diploid stem diameter, along with haploid frailty stem diameter, flag leaf length, flag leaf width, tassel length and tassel branches, exhibited low heritability and no genetic variation and were therefore excluded from further analysis (**Table 1**).

**Table 1:** Statistical summary of agronomic traits by ploidy level, haploid frailty %, and their heritability.

Trait	Ploidy & HF%	Min.	Mean	Max.	SD	95% CI	Environments (P-value)	Genotypes (P-value)	Heritability
Plant height	H	73.0	139.4	200.8	20.1	136.6-142.3	0.0	2 x 10^-16^	0.8
	D	115.5	188.7	291.7	25.0	185.1-192.3	2.5 x 10^-15^	2.2 x10^-16^	0.9
	HF%	-2%	30%	50%	10%	30%-30%	0.0	2.8 x 10^-16^	0.7
Ear height	H	15.8	43.9	80.0	12.7	42.1-45.8	1.3 x 10^-9^	2.2 x 10^-16^	0.8
	D	36.7	72.4	125.0	15.8	70.1-74.7	1.5 x 10^-15^	2.2 x 10^-16^	0.8
	HF%	-4%	40%	70%	0.1	40%-40%	0.5	1.4 x10^-15^	0.7
Stem diameter	H	2.7	4.1	17.5	1.5	3.8-4.3	0.0	0.3	0.1
	D	3.3	4.8	12.5	0.8	4.8-4.9	0.0	0.1	0.2
	HF%	-3%	20%	50%	30%	10%-20%	0.0	0.3	0.1
Flag leaf length	H	16.9	33.4	53.2	6.1	32.6-34.3	5.4 x 10^-8^	2.2 x 10^-16^	0.8
	D	19.7	33.4	53.2	6.1	32.6-34.3	3.1 x 10^-13^	2.2 x 10^-16^	0.7
	HF%	-40%	10%	50%	10%	10%-20%	0.4	0.1	0.3
Flag leaf width	H	2.4	4.4	12.3	1.0	4.2-4.5	0.0	2.2 x 10^-16^	0.7
	D	2.5	5.3	8.0	1.0	5.2-5.5	2 x 10^-11^	2.2 x 10^-16^	0.8
	HF%	-70%	20%	50%	10%	10%-20%	0.0	0.1	0.2
Tassel length	H	6.1	24.2	37.0	4.1	23.7-24.9	0.0	2 x 10^-16^	0.8
	D	10.3	30.6	46.3	4.3	30.0-31.2	8.1 x 10^-16^	2.2 x 10^-16^	0.8
	HF%	-1%	20%	80%	20%	20%-20%	0.2	0.0	0.3
Spike length	H	4.2	15.5	25.5	3.4	15.0-16.0	2.2 x 10^-5^	2.2 x 10^-16^	0.9
	D	11.2	20.0	33.5	3.4	19.5-20.5	0.0	2.2 x 10^-16^	0.9
	HF%	0%	20%	80%	0.1	20%-20%	0.2	5.7 x 10^-10^	0.6
Tassel branches	H	4.8	15.6	44.0	5.2	14.9-16.3	0.2	2 x 10^-16^	0.8
	D	6.5	16.8	36.7	5.2	16.1-17.6	0.8	2 x 10^-16^	0.8
	HF%	-60%	10%	40%	20%	0%-10%	0.5	0.3	0.1

*H, Haploids; D, Diploids; HF%, Haploid frailty percentages; Min., Minimum; Max., Maximum; SD, Standard deviation.

### Diploid-haploid pairs exhibiting high and low haploid frailty in plant height and ear height

**Figure 2:**
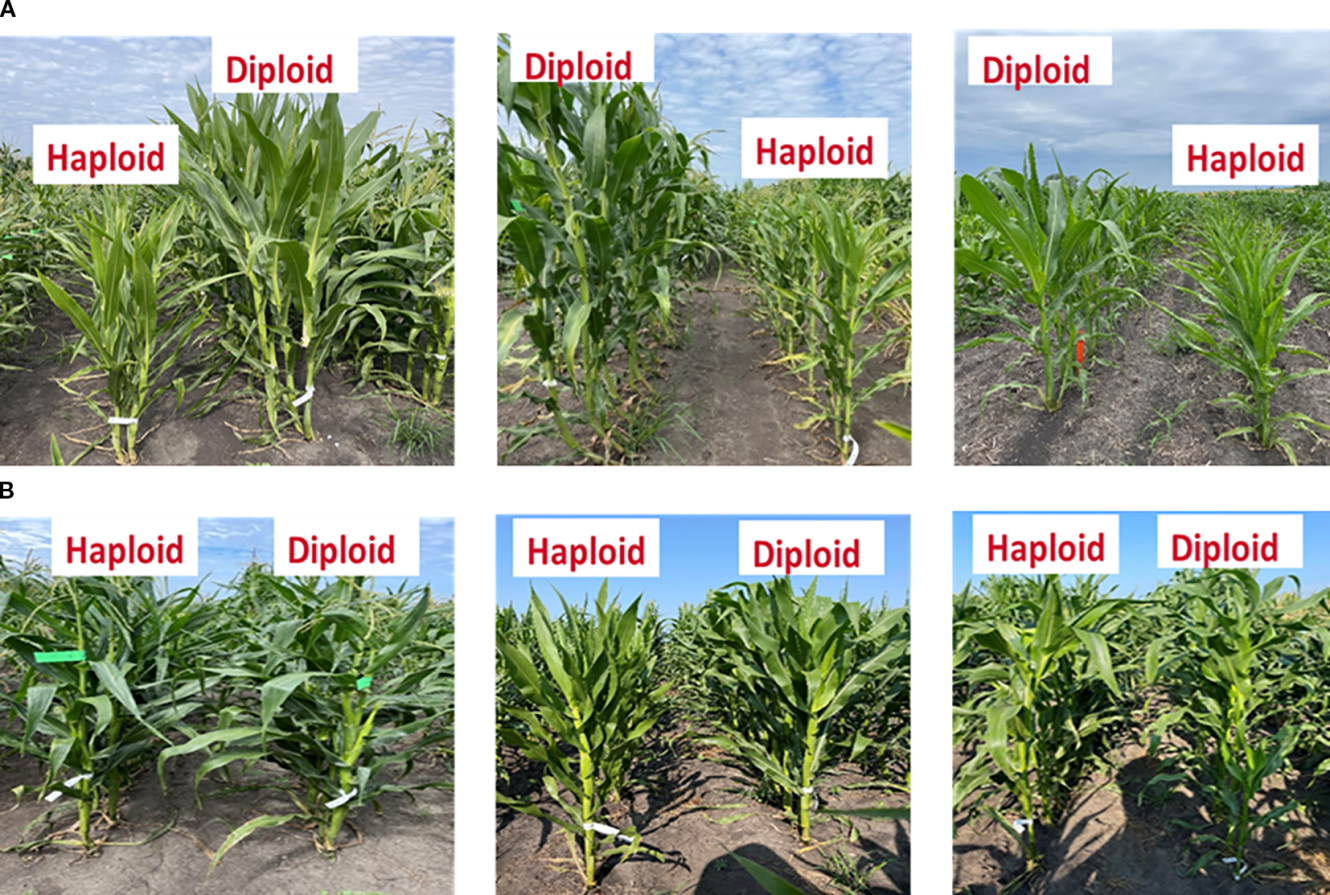
Examples of six perfect isogenic haploid and diploid line pairs. Haploids are, in most cases, shorter than corresponding isogenic diploid plants **(A)**. However, in some cases, haploid plants exhibited a height comparable to their corresponding diploids, resulting in minimal or absence of haploid frailty **(B)**.

Field observations clearly illustrated the variability in haploid frailty across isogenic diploid–haploid pairs (**Figures 2A, B**). *Panel A* shows representative pairs where haploids were consistently shorter and weaker than their diploid counterparts, demonstrating cases of high frailty. In contrast, *Panel B* highlights exceptional pairs in which haploids reached comparable stature to diploids, indicating minimal or absent frailty.

Quantitative analysis supported these observations (**Table 2**). For plant height, haploid frailty percentage (HF_PH) ranged from 0.45 to 0.77, with the SHGD_DH_211 pair exhibiting the highest frailty (HF_PH = 0.77). Similarly, for ear height, haploid frailty values (HF_EH) ranged from 0.71 to 0.86, again with SHGD_DH_211 showing the highest frailty (HF_EH = 0.86). By contrast, minimal or absent frailty was observed in certain pairs. For plant height, SHGD_DH_045 and SHGD_DH_053 showed HF_PH values of –0.02 and 0.02, respectively, indicating negligible differences between haploids and diploids. For ear height, SHGD_DH_046 displayed a negative HF_EH value (–0.04), reflecting haploids that matched or slightly outperformed diploids.

**Table 2:** Top 5 isogenic diploid-haploid line pairs with highest HF and top 5 isogenic diploid-haploid line pairs with lowest or absent HF.

Top 5 diploid-haploid lines pairs with high haploid frailty in plant height (left-hand column) and ear height (right-hand column)										
Diploid-haploid pairs	Diploid	Haploid			HF_PH	Diploid-haploid pairs	Diploid		Haploid	HF_EH
SHGD_DH_211	226.67	51.67			0.77	SHGD_DH_211	70.00		10.00	0.86
BS39_DH_111	138.33	67.00			0.52	BS39_DH_089	86.67		16.67	0.81
SHGD_DH_096	160.33	86.67			0.46	SHGD_DH_107	60.00		15.00	0.75
BS39_DH_089	230.00	126.67			0.45	BS39_DH_066	68.33		18.33	0.73
BS39_DH_040	213.33	118.33			0.45	SHGD_DH_096	81.67		23.33	0.71
Top 5 diploid-haploid lines pairs with minimal haploid frailty in plant height (left-hand column) and ear height (right-hand column)										
Diploid-haploid pairs	Diploid		Haploid	HF_PH		Diploid-haploid pairs	Diploid	Haploid		HF_EH
SHGD_DH_045	158.33		162.00	-0.02		SHGD_DH_046	40.00	41.67		-0.04
SHGD_DH_053	146.67		144.33	0.02		SHGD_DH_053	51.67	53.33		-0.03
SHGD_DH_099	157.00		147.33	0.06		SHGD_DH_139	70.00	70.00		0.00
SHGD_DH_025	157.00		146.67	0.07		SHGD_DH_067	45.00	43.33		0.04
SHGD_DH_053	151.67		141.67	0.07		SHGD_DH_103	36.67	35.00		0.05

*HF_PH, Plant height haploid frailty; HF_EH, Ear height haploid frailty.

### Correlations between haploid performance, diploid performance, and haploid frailty percentage

Correlation analysis between diploid performances, haploid performances, and haploid frailty percentages for the agronomic traits revealed significant relationships with varying degrees of strength (**Table 3**, **Supplementary Figure S3**). Correlations between diploid and haploid performance were generally positive, ranging from 0.6 for tassel length to 0.9 for number of tassel branches. Correlations between haploid performance and HF values were negative ranging from -0.4 to -0.6. Correlations between diploid performances and HF percentages are generally weaker and positive (0.1 to 0.4) for the four traits analyzed (**Table 3**, **Supplementary Figure S3**). Correlations analysis between haploid performance and haploid frailty; and diploid & haploid frailty for stem diameter, flag leaf length, flag leaf width, and the number of tassel branches were excluded due to low heritability estimates and absence of genetic variation, as shown in **Table 1**.

**Table 3:** Correlation between the performance of haploids, diploids, and haploid frailty %.

Trait	Diploid – Haploid	Haploid – HF%	Diploid – HF%
Plant height	0.8 ***	-0.5 ***	0.2 **
Ear height	0.7 ***	-0.6 ***	0.1 *
Flag leaf length	0.7 ***	n/a	n/a
Flag leaf width	0.7***	n/a	n/a
Tassel length	0.5 ***	-0.4 ***	0.4 ***
Spike length	0.8 ***	-0.6 ***	0.1 *
Tassel branches	0.9 ***	n/a	n/a

*n/a, agronomic traits excluded from correlation analysis *HF, Haploid frailty percentages; *Diploid – Haploid, Correlation of diploids and haploids; *Haploid – HF%, Correlation of haploids and haploid frailty percentages; *Diploid-HF%, Correlation of diploids and haploid frailty percentages; asterisk, P-value significance (<0.05, <0.01, <0.001).

### Correlations of haploid female fertility, haploid male fertility, with agronomic traits and haploid frailty percentage

**Figure 3:**
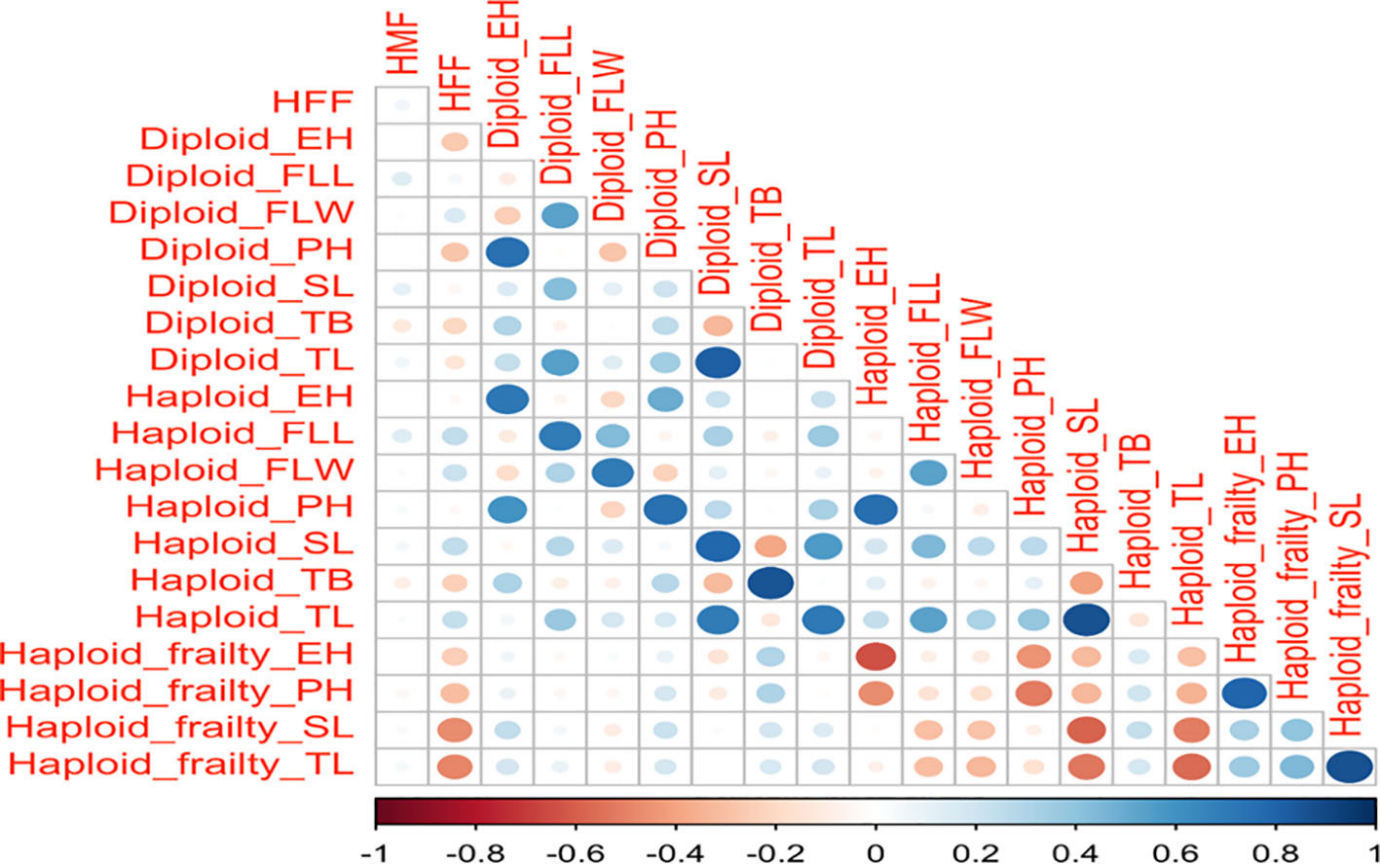
Correlation matrix of HFF, HMF, and the eight agronomic traits across haploids, diploids, and haploid frailty percentages. The color gradient, with blue indicating positive correlations and red indicating negative correlations, along with the size of the circles, represents the strength of these correlations. HMF, Haploid male fertility; HFF, Haploid female fertility; PH, Plant height; EH, Ear height; SD, Stem diameter; FLL, Flag leaf length; FLW, flag leaf width; TL, Tassel length; SL, spike length and TB, number of tassel branches.

**Figure 3** illustrates the relationship between haploid male fertility, haploid female fertility and agronomic traits across diploid, haploid, and haploid frailty percentages. Haploid male fertility showed a correlation of 0.3 for haploid flag leaf length and diploid flag leaf length, and 0.2 for diploid spike length, tassel length and tassel branches. Haploid female fertility demonstrated slightly stronger correlations than haploid male fertility, with a correlation of 0.4 for haploid flag leaf length, flag leaf width, spike length and tassel length and 0.3 for diploid flag leaf width.

### Predictive ability of diploids for haploid and haploid frailty percentage

**Figure 4:**
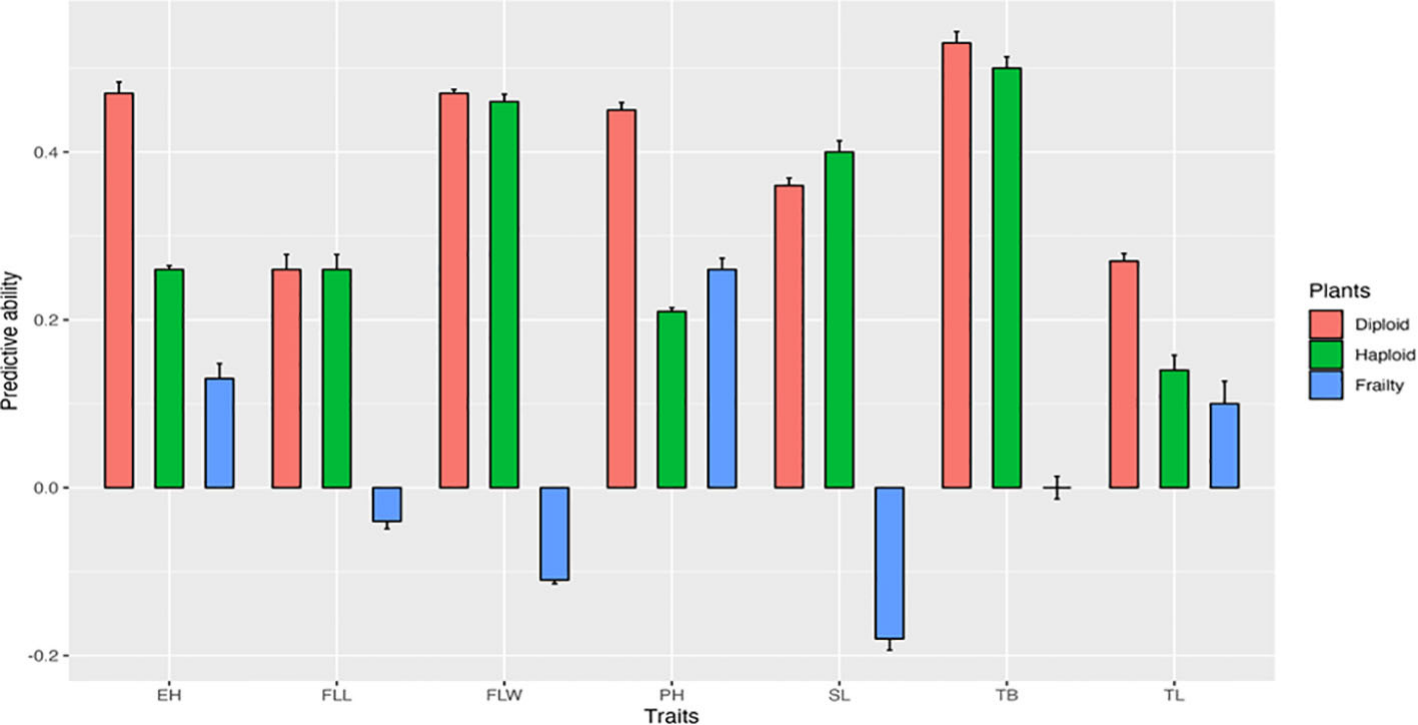
Genomic predictive abilities of diploid performance for haploid performance, and haploid frailty across seven agronomic traits. PH, Plant height; EH, Ear height; FLL, Flag leaf length; FLW, Flag leaf width; SL, Spike length; TB, Number of tassel branches; TL, Tassel length.

**Figure 4** shows the predictive ability of diploids for haploid performance and haploid frailty across the seven traits. Diploids generally displayed moderate predictive ability (0.3–0.5) for most haploid traits, suggesting that diploid performance can provide some insight into haploid performance. However, predictive ability was weaker for haploid frailty, particularly for flag leaf length, flag leaf width, and spike length, where values were close to zero.

### Association mapping for haploid performance, diploid performance, and haploid frailty percentages

Genome-wide association analysis revealed distinct SNP associations for diploid performance, haploid performance, and haploid frailty across agronomic traits (**Table 4**; **Supplementary Figure S4**). For plant height, significant loci were identified on chromosomes 3 and 10 for diploid performance, on chromosomes 2 and 3 for haploids, and on chromosomes 1 and 8 for haploid frailty. For ear height, 12 SNPs on chromosomes 3 and 8 were detected for diploid performance while 15 SNPs on chromosomes 1, 2, 3, 4, 5, 8 and 10 were detected for haploid performance with highly significant associations on chromosome 1 (P = 1 × 10^-15^) and chromosome 2 (P = 1 × 10^-^¹²) for haploid performance. Three SNPs on chromosome 4. 9, and 10 were associated with haploid frailty ear height. Flag leaf traits showed multiple associations: 13 SNPs for diploid flag leaf length (chromosomes 1, 3, 6, 7) and seven SNPs for haploids (chromosomes 1, 2, 7). Haploid flag leaf width was strongly associated with 17 SNPs across seven chromosomes, including a highly significant locus on chromosome 3 (P = 1 × 10^-^¹²), whereas diploids showed only two SNPs (chromosomes 1, 6). For spike length, one SNP was identified for diploids (chromosome 9), and one for haploid frailty (chromosome 4), while haploid performance showed no significant associations. Tassel traits exhibited broader associations: diploid tassel length was linked to 10 SNPs (chromosomes 2, 4), haploid tassel length to six SNPs (chromosomes 1, 6), and haploid frailty to seven SNPs (chromosomes 1, 4, 5, 7, 9). For tassel branch number, three SNPs were associated with diploid performance (chromosome 5) and three with haploid performance (chromosomes 1, 5, 8), including a shared SNP on chromosome 5. Overall, only one SNP overlapped between haploid and diploid tassel branch number. No SNPs were common across diploid performance and haploid frailty, or between haploid performance and haploid frailty, for any of the evaluated traits.

**Table 4:** Significant SNPs detected for diploid performance, haploid performance, haploid frailty percentages, and common SNPs/regions across diploid performance and haploid performance.

Trait	Diploids (Chr)	Haploids (Chr)	Haploid frailty (Chr)	Shared SNPs (D–H)
Plant height	4 (Chr 3, 10)	5 (Chr 2, 3)	5 (Chr 1, 8)	None
Ear height	12 (3, 8)	15 (Chr 1, 2, 3, 4, 5, 8,10)	3 (Chr 4, 9, 10)	None
Flag leaf length	13 (Chr 1, 3, 6, 7)	7 (Chr 1, 2, 7)	n/a	None
Flag leaf width	2 (Chr 1, 6)	17 (Chr 1, 2, 3, 6, 7, 8, 9)	n/a	None
Tassel length	10 (Chr 2, 4)	6 (Chr 1, 6)	7 (Chr 1, 4, 5, 7, 9)	None
Spike length	1 (Chr 5)	0	1 (Chr 4)	None
Tassel branches	3 (Chr 5)	3 (Chr 1, 5, 8)	n/a	1 (Chr 5)

*Bolded numbers indicate significant SNPs detected across diploid performance and haploid performance; n/a, No GWAS was conducted for the haploid frailty percentages of these traits since they are not heritable; D-H, SNPs common across diploid performance and haploid performance.

### Candidate gene mapping of highly significant SNPs and SNPs overlapping across haploids and diploids

Several candidate genes were identified as being in LD with highly significant SNPs for haploid performance and SNPs common across haploid performance and diploid performance Supplementary (**Supplementary Table S1**, **Supplementary Figure S4**). For the number of tassel branches, a common SNP was identified across haploid performance and diploid performance. The SNP S5_135455228 on chromosome 5 is in LD with Zm00001eb237460, encoding a Myb-related protein 3R-1. The highly significant SNP (S5_60244705) on chromosome 5 is associated with Zm00001eb227800, encoding Protein SHI RELATED SEQUENCE 1. Two SNPs were associated with ear height. SNP S1_267783859 on chromosome 1 is linked to Zm00001eb054870, which encode a SAUR-like auxin-responsive protein and SNP S2_197433320 on chromosome 2 is associated with Zm00001eb104060, encoding a PRONE domain-containing protein. One candidate gene was in LD with SNPs associated with haploid performance of flag leaf width, SNP S3_6432571 on chromosome 3 is next to Zm00001eb121230, encoding Homogentisate geranylgeranyl transferase1 (**Supplementary Table S1**).

## Discussion

### Historical perspective and recent studies

The concept of haploid frailty (HF) was first described by Chase (1964), who observed that maize haploids were smaller and less vigorous than expected under the “haploid–diploid ratio” model. This foundational work established frailty as a biological constraint rather than a simple scaling effect. More recently, Yavuz et al. (2025) quantified HF in BS39-derived populations and reported that the *qshgd1* locus reduces frailty across multiple traits, confirming that HF is both genetically variable and heritable. In parallel, Fakude et al. (2025) investigated haploid fertility in a BS39-derived haploid isogenic lines and showed that haploid male fertility and haploid female fertility are genetically independent traits, each with distinct genetic architectures. Together, these studies clarified the heritability of HF and the genetic independence of haploid fertility.

Our study extends these findings by integrating haploid frailty, haploid vigour, and haploid fertility traits with correlation analysis, genomic prediction and genome-wide association analysis. We focus here on what is novel: the identification of vigorous haploids (cases of negative frailty), correlations linking haploid vigour with haploid fertility, and the discovery of haploid-specific loci and candidate genes that provide functional insight into haploid biology. Importantly, this study also evaluates the possibility of breeding haploids that are both vigorous and fertile, which, if realized, could transform doubled haploid breeding strategies.

### Novel insights into haploid frailty

Consistent with Chase (1964) and Yavuz et al. (2025), haploids in our study exhibited 30–40% reductions in plant and ear height compared to diploids. Yet, we also detected traits with minimal or even negative frailty, such as flag leaf width and tassel branch number, where haploids sometimes equaled or outperformed diploids. These observations demonstrate that frailty is trait-specific and variable, opening opportunities to select for inherently vigorous haploids. Notably, heritability estimates confirmed that HF is not purely environmental: values of 0.6–0.7 for plant height, ear height, and spike length support the feasibility of genetic improvement for haploid vigour.

### Exclusion of low-heritability traits from further analysis

Heritability and genetic variance were evaluated for all eight traits. Stem diameter was excluded in both diploids and haploids due to low variance and heritability, which likely reflects its developmental plasticity and high environmental sensitivity (Li et al., 2018). Because haploid frailty is derived from haploid and diploid performance, low baseline heritability in both states also resulted in low frailty heritability. Haploid frailty values for flag leaf length, flag leaf width, and tassel branch number were similarly excluded. Although these traits showed high heritability individually, the minimal mean differences between haploids and diploids centered frailty values near zero, yielding insignificant heritability. The lack of significant genotype-by-ploidy interaction further suggested these traits are insensitive to ploidy changes and therefore less informative for assessing frailty. As a result, stem diameter was removed entirely, while only the frailty values for flag leaf traits and tassel branch number were excluded from downstream correlation, genomic prediction, and GWAS analyses. This approach aligns with prior work showing that traits with higher heritability are more responsive to selection (Falconer and Mackay, 1996; Hallauer et al., 2010), whereas traits with low heritability are unlikely to be improved effectively through genetic selection.

### Heritability of diploid performance, haploid performance and haploid frailty

High heritability values in diploids (0.8 to 0.9) indicate strong genetic control, suggesting that selective breeding in diploids is effective due to minimal environmental influence (Tuhina-Khatun et al., 2015). Similar results have been observed in other maize studies, with plant height and ear height showing heritability >0.8 (Michel et al., 2022; Yang et al., 2024) and flag leaf length and flag leaf width showing heritability >0.6 (Li et al., 2015). Similarly, high but slightly lower heritability (0.7 to 0.9) in haploids suggests a good potential for genetic improvement. The slight reduction of heritability for haploid compared to diploid performances could be caused by the effect chimerism. Since haploid plants may carry varying fractions of diploid cells, this may introduce additional variability, reducing the genetic contribution to trait expression on genetic consistency. In contrast, haploid frailty exhibited lower heritability (0.1 to 0.7). This could be due to haploid frailty being a calculated trait, derived from two separate traits (i.e., haploid and diploid performance), each with its own variation. When these traits are combined, the overall variation is amplified, leading to lower heritability estimates.

### Correlations between haploid vigour and haploid fertility traits

A key novel contribution of this work is the analysis of correlations between HF, HMF, and HFF. While Fakude et al. (2025) established that HMF and HFF are independent, they did not examine their relationships with vigour traits. Here, we show that HFF correlates positively with haploid frailty traits (flag leaf length, flag leaf width, tassel length, and spike length), and HMF shows a positive but weak correlation with flag leaf length, tassel length, spike length and tassel branches. These findings suggest that breeding for haploid vigour could indirectly improve fertility, particularly HFF, thereby enhancing DH line recovery. This opens the possibility of simultaneously improving haploid vigour and fertility, allowing the development of haploids that are not only robust but also reproductively competent. Such a dual improvement strategy would enhance the efficiency of DH pipelines.

### Genomic prediction and breeding applications

The goal of this analysis was to assess whether diploid data could predict haploid performance and haploid frailty across seven agronomic traits. Diploid models showed weak to moderate predictive ability (0.3–0.5) for traits such as ear height, plant height, and tassel branch number, suggesting that diploid data can be useful proxies in these cases. Predictive ability was weaker for traits like flag leaf length and tassel length, while haploid frailty predictions were generally negligible, with some traits showing near-zero or negative values (e.g., flag leaf width at –0.11 and spike length at –0.18). These results indicate that frailty-specific variation is not fully captured by diploid data, emphasizing the need for direct haploid evaluations. Incorporating haploid performance into genomic selection schemes will therefore be essential for reducing frailty and improving the efficiency of doubled haploid pipelines.

### Consistency with prior agronomic GWAS

For plant height, diploid associations were detected on chromosomes 3 and 10, haploid associations on chromosomes 2 and 3, and haploid frailty associations on chromosome 8. Previous GWAS consistently implicated chromosomes 1 and 2 in height regulation (Wang et al., 2025), while Wen et al. (2025) identified stable SNPs on chromosomes 8 and 10. Collectively, these results emphasize chromosomes 1, 2, 8, and 10 as robust regulators of maize height across ploidy levels. For ear height, the strongest haploid signals occurred on chromosomes 1 and 2, consistent with QTL reported in linkage and association studies (Peiffer et al., 2014; Chen et al., 2012), confirming the central role of these regions in controlling plant stature. For flag leaf traits, diploid associations for leaf length localized to chromosomes 1, 3, 6, and 7, whereas haploid associations mapped to chromosomes 1, 2, and 7. For leaf width, diploid associations were limited to chromosomes 1 and 6, but haploid associations extended across seven chromosomes (1, 2, 3, 6, 7, 8, and 9). These results mirror large-scale GWAS linking leaf morphology to chromosomes 1, 3, 6, and 7 (Zeng et al., 2025), underscoring their role as conserved regulators of leaf architecture across genetic backgrounds and ploidy states. For tassel traits, clear ploidy-specific differences emerged. Diploid tassel length mapped to chromosomes 2 and 4, while haploid tassel length mapped to chromosomes 1 and 6. Haploid frailty associations were more dispersed, spanning chromosomes 1, 4, 5, 7, and 9, suggesting a broader genetic basis for tassel development under haploid conditions.

### Candidate gene mapping and functional implications

Candidate gene mapping of highly significant SNPs and overlapping loci across haploids and diploids revealed biologically meaningful associations that link genetic variation with both trait expression and practical breeding potential. For tassel branch number, one shared SNP (S5_135455228) was detected across haploid and diploid performance. This SNP is in LD with Zm00001eb237460, encoding Myb-related protein 3R-1, a transcription factor that regulates the G2/M cell cycle transition. In Arabidopsis, disruption of Myb3R impairs cytokinesis and gametophyte development (Haga et al., 2007; Kobayashi et al., 2015). Notably, Fakude et al. (2025) also identified this gene as associated with haploid female fertility in field corn, indicating its pleiotropic role in both vegetative development and reproductive success. From a breeding perspective, Myb3R-1 represents a particularly valuable target because markers linked to this gene could enable simultaneous selection for haploid vigour and fertility, reducing the need for artificial doubling and improving recovery rates in DH pipelines.

In addition, a diploid-specific highly significant SNP (S5_60244705) was associated with Zm00001eb227800, encoding SHI-RELATED SEQUENCE 1 (SRS1). This gene regulates auxin biosynthesis and organ elongation (Gomariz-Fernández et al., 2017), processes that influence plant architecture. Its significance in diploids but not haploids suggest ploidy-dependent regulation of tassel branching. While its relevance for haploid improvement may be limited, this gene provides breeders with an avenue to refine tassel architecture in diploid breeding schemes.

Two additional SNPs were associated with ear height. The first, S1_267783859 on chromosome 1, was linked to Zm00001eb054870, which encodes a SAUR-like auxin-responsive protein. SAUR family members are among the earliest responders to auxin signalling, promoting cell elongation and tissue expansion (Ren and Gray, 2015; Stortenbeker and Bemer, 2019). The identification of this locus in association with ear height indicates the role of auxin-mediated internode elongation. For breeders, this implies that SAUR-linked markers could be used to select haploids with improved stature and optimal ear placement, traits that strongly influence plant performance and yield potential. The second SNP, S2_197433320 on chromosome 2, was associated with Zm00001eb104060, a PRONE domain–containing protein that functions as a ROP-GEF. These proteins regulate Rho-like GTPase signalling, cytoskeletal organization, and morphogenesis (Feiguelman et al., 2018). By influencing cell division and tissue expansion, this locus provides a mechanistic basis for variation in plant stature and ear height. From a practical standpoint, markers linked to PRONE-domain proteins could be used to improve stem architecture and standability, thereby reducing lodging risk in field environments.

Finally, for flag leaf width, SNP S3_6432571 on chromosome 3 was linked to Zm00001eb121230, encoding homogentisate geranylgeranyl transferase 1 (HGGT1), an enzyme that catalyzes the first committed step in tocopherol (vitamin E) biosynthesis (Niu et al., 2022). Tocopherols are vital antioxidants that stabilize membranes and protect against oxidative stress. The association of HGGT1 with leaf morphology suggests that metabolic resilience and stress tolerance contribute to haploid vigour. For breeders, this gene represents a promising entry point for selecting haploids that not only perform well under optimal conditions but also maintain vigour under abiotic stress, thereby improving field adaptation.

Collectively, these candidate genes show functional pathways in auxin signalling (SRS1, SAUR), cell cycle and cytoskeletal regulation (Myb3R-1, PRONE), and oxidative stress protection (HGGT1). By tying SNP-trait associations to these mechanistic pathways, this study provides breeders with tangible targets for marker-assisted selection and genomic prediction. Notably, Myb3R-1 emerges as a consensus pleiotropic regulator across haploid and diploid contexts, while auxin- and stress-related pathways offer complementary strategies for breeding haploids that are both vigorous and fertile.

## Conclusions

This study advances the understanding of haploid biology in maize by integrating analyses of haploid frailty, and haploid fertility with genomic prediction and association mapping. We demonstrate that haploid frailty is heritable, trait-specific, and in some cases even absent, revealing the existence of inherently vigorous haploids with breeding potential. Correlations between haploid frailty and haploid fertility, suggest that both traits can be improved simultaneously, opening pathways toward haploids that are robust and reproductively competent. While diploid data offered only moderate predictive ability for haploid traits, direct evaluation of haploids remains essential for effective selection. Candidate gene mapping highlighted functional regulators including Myb3R-1, SRS1, SAUR-like auxin-responsive proteins, PRONE-domain proteins, and HGGT1, implicating pathways in cell cycle control, auxin signaling, and oxidative stress protection. These loci provide breeders with tangible targets for marker-assisted selection, genomic prediction, and functional validation. Collectively, our findings demonstrate the feasibility of breeding haploids that are both vigorous and fertile, a strategy that could transform doubled haploid pipelines by reducing reliance on artificial doubling and enhancing the efficiency of maize breeding programs.

## Data Availability

The datasets presented in this study are publicly available. This data can be found here: https://github.com/Mercyfakude/Data-and-code-availability.

## Supplementary material

Supplementary Figure 1 Minimum Haploid frailty percentages across traits.

Supplementary Figure 2 Haploid frailty heritability estimates across traits.

Supplementary Figure 3 Visualization of correlation analyses.

Supplementary Table 1 Functional annotations of candidate genes associated with highly significant SNPs and SNPs common across haploid performance, diploid performance and haploid frailty %.

Supplementary Figure 4 Stacked Manhattan plots for haploid performance, diploid performance, and haploid frailty percentage across 7 traits.
