# Supplementary material for: Dissecting the genetic variation of haploid frailty in maize for enhanced doubled haploid breeding
Source: Front Plant Sci. 2025 Oct 7;16:1646128. doi: 10.3389/fpls.2025.1646128 (PMC12537748; doi:10.3389/fpls.2025.1646128)
Supplement: Supplementary Figure 1 — Minimum Haploid frailty percentages across traits. [file DataSheet1.pdf]

SUPPLEMENTARY MATERIAL

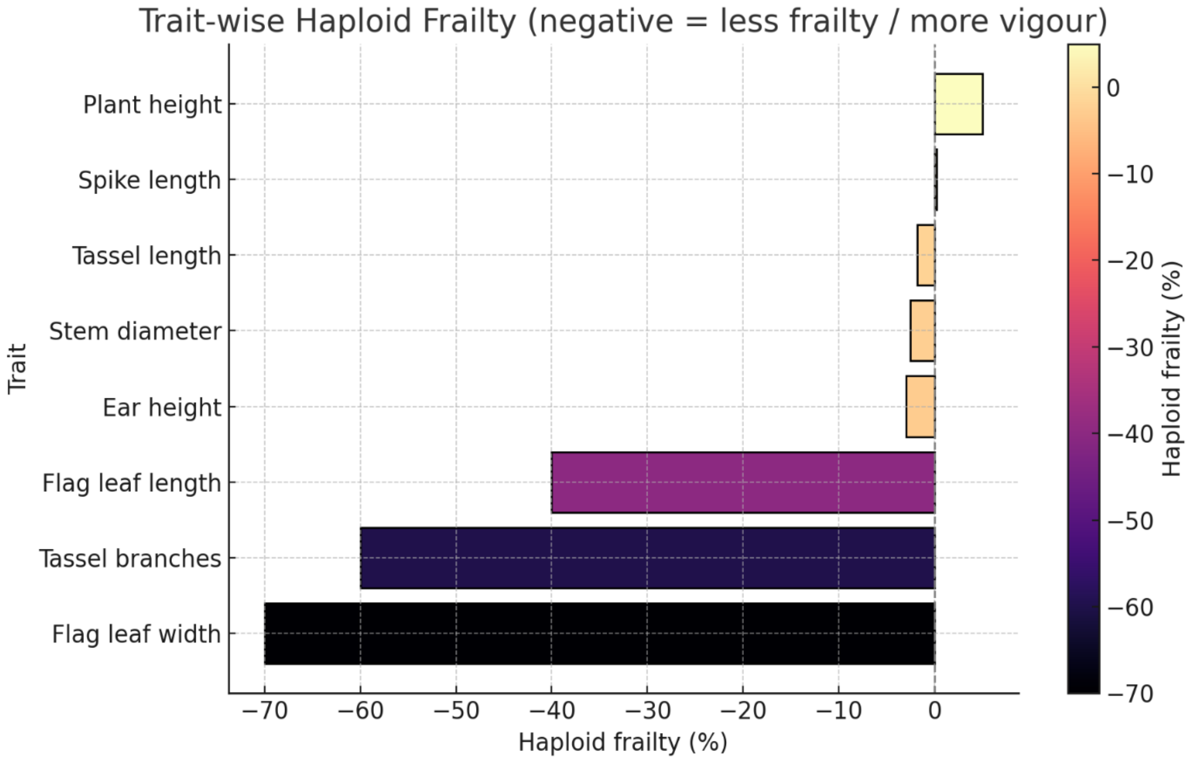

Supplementary Figure S1 Minimum Haploid frailty percentages across traits as shown in Table 1. PH = Plant height; EH = Ear height; FLL = Flag leaf length; FLW = Flag leaf width; SL = Spike length; TB = Number of tassel branches; TL = Tassel length.

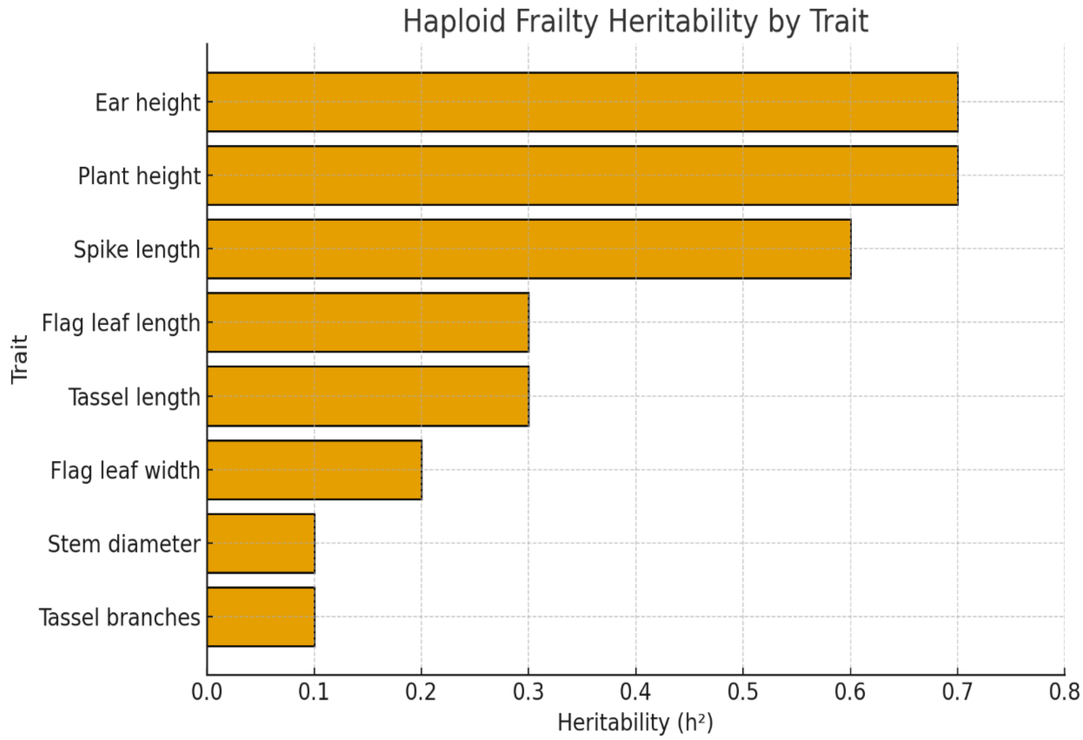

Supplementary Figure S2 Haploid frailty heritability estimates across traits as shown in Table 1. PH = Plant height; EH = Ear height; FLL = Flag leaf length; FLW = Flag leaf width; SL = Spike length; TB = Number of tassel branches; TL = Tassel length.

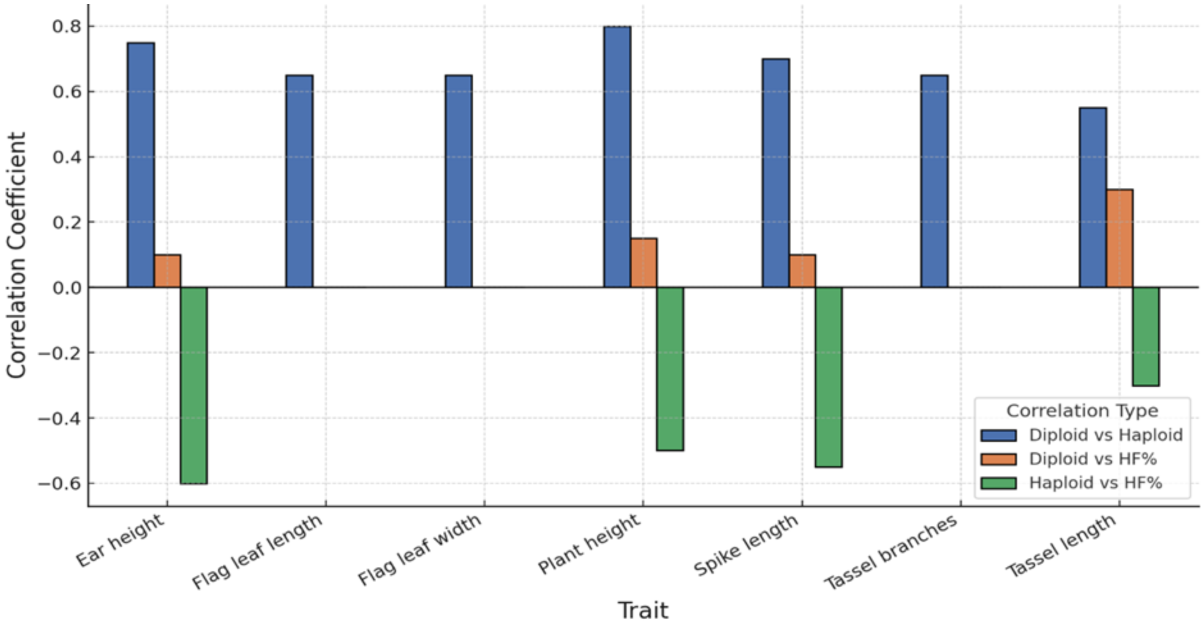

Supplementary Figure S3 Visualization of correlation analyses as shown in Table 3. Correlation between diploid performance and haploid performance; Correlation between diploid performance and haploid frailty percentages; Correlation between haploid performance and haploid frailty percentages.

Supplementary Table S1 Functional annotations of candidate genes associated with highly significant SNPs and SNPs common across haploid performance, diploid performance and haploid frailty %

Trait	SNP	Overlap/Highly significant	Ploidy	Candidate genes	Position	Functional annotation
**TB**	S5_135455228	Overlapping	H and D	Zm00001eb237460	chr5:137986868 - 138015382	Myb-related protein 3R-1
	S5_60244705	Highly Significant	D	Zm00001eb227800	chr5:61996357 - 61999172	Protein SHI RELATED SEQUENCE 1
**EH**	S1_267783859	Highly Significant	H	Zm00001eb054870	chr1:274141496 - 274142102	SAUR-like auxin-responsive protein family
	S2_197433320	Highly Significant	H	Zm00001eb104060	chr2:203405080 - 203408547	PRONE domain-containing protein
**FLW**	S3_6432571	Highly Significant	H	Zm00001eb121230	chr3:6527570 - 6529519	Homogentisate geranylgeranyl transferase1

* *TB =Number of Tassel branches; EH= Ear height; FLW= flag leaf width; H= Haploids; D= Diploids; HF=Haploid frailty; chr=Chromosome*

Supplementary Figure S4 Stacked Manhattan plots for haploid performance, diploid performance, and haploid frailty percentage across 7 traits

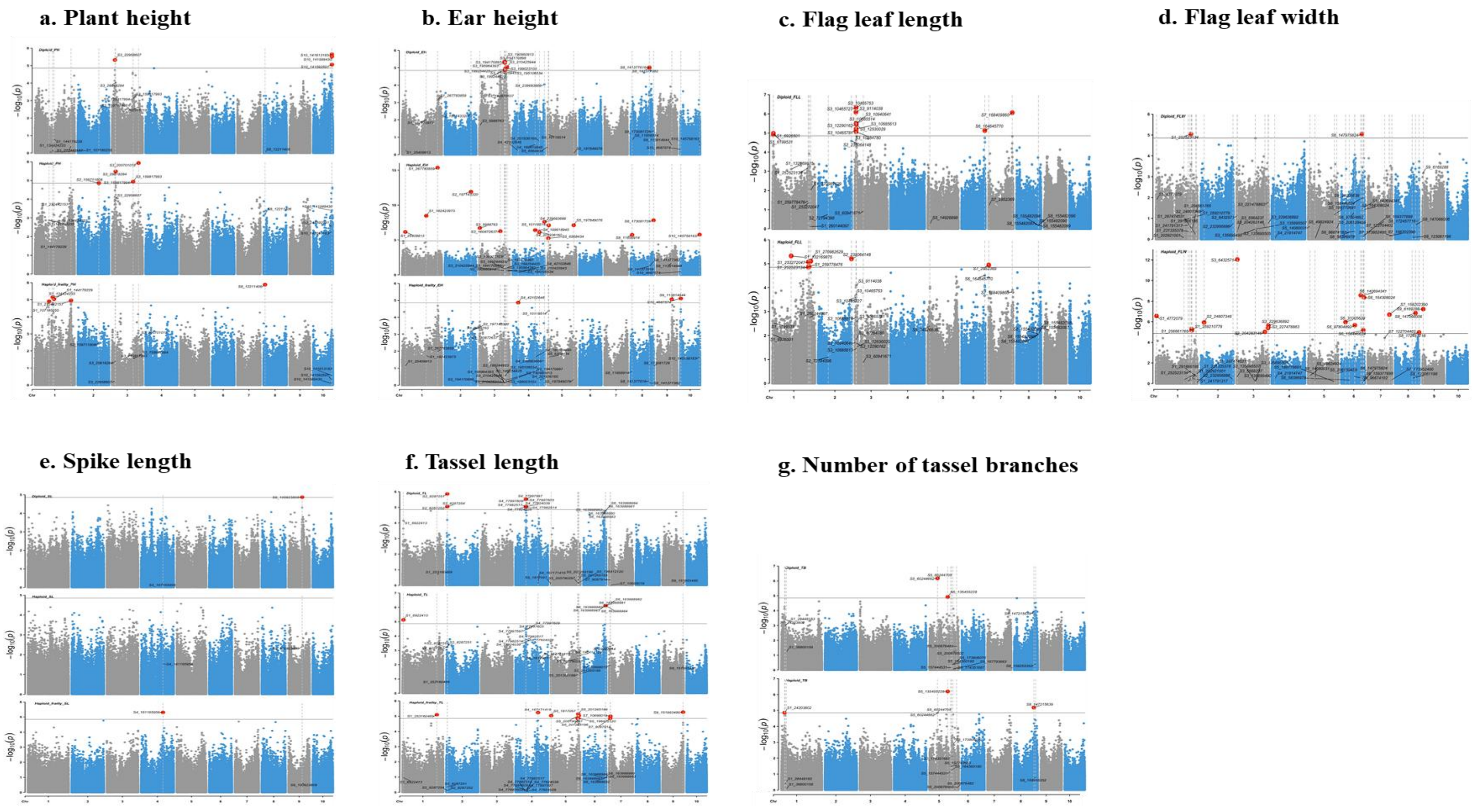

Supplementary Figure S4 Manhattan plots of diploids, haploids, and haploid frailty values using the FARMCPU. Manhattan plots (a-g) show association mapping of diploid and haploid performance, as well as haploid frailty across four agronomic traits (PH, EH, TL, SL) and haploid and diploids performance of FLL, FLW, TB without haploid frailty as they were excluded from analysis due to lack of genetic variation and low heritability. The red dots above the black line represent significant SNPs. The black line represents the significance threshold. The Y-axis represents the –log10 P-value, and the X-axis represents the chromosomes.
